# Comparative analysis of short answer questions and multiple-choice questions in formative assessment of first-year medical students

**DOI:** 10.1186/s12909-026-10344-1

**Published:** 2026-09-23

**Authors:** Asmaa M. Ammar, Heba M. El Naggar, Manal B. Ahmed, Zeinab Elsayed, Gihan M. Tawfeek

**Affiliations:** 1 Medical Parasitology Department, Faculty of Medicine, Ain Shams University, Cairo, 1181 Egypt; 2 Medical Biochemistry and Molecular Biology Department, Faculty of Medicine, Ain Shams University, Cairo, 1181 Egypt; 3 Clinical Oncology Department, Faculty of Medicine, Ain Shams University, Cairo, 1181 Egypt; 4 Medical Education Department, Faculty of Medicine, Ain Shams University, Cairo, Egypt

**Keywords:** Multiple-choice questions, Short answer questions, Psychometric analysis, Item difficulty, Item discrimination

## Abstract

**Background:**

Assessment methods are essential for evaluating medical students’ knowledge and cognitive skills. Multiple-choice questions (MCQs) are widely used because of their objectivity and efficiency, whereas short answer questions (SAQs) are considered better suited for assessing higher-order cognitive skills. However, evidence comparing the psychometric performance of these formats in formative undergraduate medical assessments remains limited. This study aimed to compare the difficulty index (P), discrimination index (DI), and overall test performance of SAQs and MCQs in a formative assessment of first-year medical students.

**Methods:**

A descriptive analytical cross-sectional study was conducted among 178 first-year medical students enrolled in the Medical Parasitology course within the Locomotor and Skin module at Ain Shams University during the 2024–2025 academic year. Students completed content-matched SAQ and MCQ examinations, each comprising 20 items aligned with the same intended learning outcomes. Psychometric analyses included item difficulty, discrimination, reliability, and comparisons between formats.

**Results:**

Students achieved significantly higher scores on MCQs than SAQs (13.5 vs. 11.1/20, *p* < 0.001). MCQ items were significantly easier (mean p: 0.67 vs. 0.55, *p* < 0.001), while discrimination indices were comparable (0.44 vs. 0.41, *p* = 0.398). Both formats demonstrated excellent internal consistency (Cronbach’s α = 0.85 for MCQs and 0.83 for SAQs) and strong discrimination between high- and low-achieving students. Pass/fail concordance was 68.0%, with more students passing MCQs but failing SAQs (*p* = 0.012). Item difficulty correlated strongly across formats (*r* = 0.71), whereas discrimination showed a weak, non-significant correlation (*r* = 0.01). Clinical SAQs were associated with lower performance among low-achieving students.

**Conclusions:**

Both MCQs and SAQs demonstrated sound psychometric properties. MCQs were easier and yielded higher scores, whereas SAQs provided a more challenging assessment with comparable discrimination. Using both formats together may enhance assessment quality in undergraduate medical education.

**Supplementary Information:**

The online version contains supplementary material available at https://doi.org/10.1186/s12909-026-10344-1.

## Background

Assessment is a cornerstone of medical education, serving not only as a tool for measuring students’ knowledge and skills but also as a powerful driver of learning. The principle that “assessment drives learning” underscores how assessment formats influence students’ study strategies, depth of understanding, and cognitive engagement. In competency-based medical education, assessment systems are increasingly expected to evaluate not only factual knowledge but also higher-order cognitive processes such as application, analysis, and clinical reasoning [1].

Written assessments remain widely used in undergraduate medical education due to their feasibility, standardization, and scalability. Among these, multiple-choice questions (MCQs) and short-answer questions (SAQs) are the most commonly used formats. MCQs, particularly single-best-answer items, are valued for their objectivity, reliability, and efficiency in scoring, especially when large cohorts are assessed [2]. Advances in item-writing guidelines and blueprinting have enabled MCQs to assess higher cognitive levels when well constructed, such as through clinical vignettes and application-based stems [3].

In contrast, SAQs are often perceived as better suited for assessing deeper understanding, synthesis of information, and recall without cueing. By requiring learners to generate responses rather than recognize correct options, SAQs may reduce guessing and cueing effects commonly associated with MCQs [4]. However, SAQs are more time-consuming to score, and concerns remain regarding subjectivity, inter-rater variability, and feasibility in large classes.

From a psychometric perspective, the quality of an assessment is determined by its reliability, validity, difficulty index, and discrimination index. The difficulty index reflects how challenging an item is for the tested cohort, while the discrimination index indicates how effectively an item differentiates between high- and low-performing students. These indices are central to post-examination item analysis and are essential for continuous improvement of assessment quality [5]. High-quality assessment items should ideally demonstrate moderate difficulty and strong discrimination, regardless of the format used.

Several studies have compared MCQs and SAQs in terms of student performance and reliability. Many have reported higher mean scores in MCQs compared to SAQs, suggesting that MCQs may be easier for students or more susceptible to cueing effects [6, 7]. Conversely, some research indicates that SAQs may show superior discrimination, particularly for higher-achieving students, due to their demand for constructed responses and integrative thinking [8]. Despite these findings, results across studies remain inconsistent, partly due to variations in item quality, content coverage, cognitive level, and assessment context.

An important limitation in the existing literature is that many comparative studies evaluate MCQs and SAQs drawn from different content areas or aligned with different learning objectives. Such designs confound the effect of question format with differences in content difficulty and cognitive demand. Additionally, relatively few studies have examined both formats within the same examination blueprint, mapped to identical intended learning outcomes and cognitive levels. This gap limits the ability to draw definitive conclusions about the inherent psychometric strengths and weaknesses of each format.

Furthermore, most available studies focus on summative assessments, where high stakes may influence student motivation and test-taking behavior. In contrast, formative assessments — designed primarily to support learning rather than grading — have received less attention in psychometric comparison studies. Yet formative assessments play a critical role in early medical education by providing feedback, identifying learning gaps, and guiding instructional planning [9]. Understanding how different assessment formats perform psychometrically in formative contexts is therefore essential.

Another underexplored area is the differential performance of assessment items across student achievement levels. While discrimination indices provide a global measure of item effectiveness, fewer studies have explicitly compared how MCQs and SAQs differentiate between high- and low-achieving students within the same cohort. Such analyses are particularly relevant in formative assessment, where the goal is to identify struggling learners while also challenging high performers.

In the context of integrated, modular medical curricula — such as the 5 + 2 undergraduate medical program adopted by many institutions — assessment strategies must align with multidisciplinary learning outcomes and increasing emphasis on clinical reasoning from early stages. Courses like Medical Parasitology, traditionally perceived as fact-heavy, are now expected to incorporate clinical vignettes and application-based questions to foster meaningful learning. Evaluating whether SAQs or MCQs are more effective in discriminating cognitive performance within such courses has direct implications for assessment design and curriculum quality assurance.

The rationale for the present study stems from the need to generate evidence-based guidance for assessment design in formative medical education. By comparing MCQs and SAQs constructed from the same blueprint, aligned with the same intended learning outcomes, and administered to the same cohort under standardized conditions, this study aims to isolate the effect of question format on difficulty, discrimination, and overall assessment quality. The findings are expected to inform educators and curriculum planners on the optimal use and integration of MCQs and SAQs to enhance the validity, fairness, and educational impact of formative assessments.

## Methods

### Study design and time period

A descriptive, analytical, cross-sectional study was conducted between February and May 2025 to compare the psychometric properties of Short Answer Questions (SAQs) and Multiple-Choice Questions (MCQs) used in the formative assessment of first-year medical students.

### Study setting and population

The study was undertaken in the Medical Parasitology Department, Faculty of Medicine, Ain Shams University, Egypt, during the Locomotor and Skin (MLS-1) module. The undergraduate medical curriculum at Ain Shams University follows the 5 + 2 curriculum (2024 bylaws), consisting of two and a half preclinical years followed by two and a half clinical years. The MLS-1 module is delivered during the second semester of the first academic year and carries 12 credit hours. The Medical Parasitology component comprises seven theoretical lectures and five practical sessions. Student assessment throughout the module includes two continuous assessments (30% of the total module score) and a final written examination consisting of MCQ and SAQ components contributing 60% and 40% of the written examination score, respectively. The study’s formative exam was conducted electronically in the Faculty’s electronic examination hall using the Qorrect^®^ e-assessment platform.

### Participants

A cluster random sample of 187 students was selected from approximately 500 Egyptian first-year medical students enrolled in the MLS-1 module during the second semester of the 2024–2025 academic year. Eligible participants were first-year students registered in the MLS-1 module under the 5 + 2 curriculum, attending the formative assessment session, and providing informed consent. Students who failed to complete both assessment formats (MCQ and SAQ) or withdrew from the course during the study period were excluded.

The formative assessment was a scheduled, curriculum-integrated component of the practical section of the parasitology course in the locomotor module and was announced two weeks in advance. All students enrolled in the module were expected to participate. The assessment served as a comprehensive review of the module immediately before the high-stakes final examination.

### Assessment development

Two content-matched examinations, each consisting of 20 SAQs and 20 MCQs, were developed using the same assessment blueprint (Supplementary File 1). Both examinations were mapped to the same learning outcomes (LOs) to ensure direct comparison between assessment formats. Based on Bloom’s taxonomy, 13 questions in each examination assessed lower-order cognitive skills (remembering and understanding), whereas 7 assessed higher-order cognitive skills through application and problem-solving. Question formats included clinical vignette-based scenarios, laboratory interpretation, comparative analysis, application of theoretical knowledge, and case-based reasoning. Items were systematically classified as factual knowledge or clinical-vignette-based through expert panel consensus during the blueprint development phase, ensuring strict alignment with the intended learning outcomes and cognitive levels.

In this study, SAQs were constructed-response items requiring students to generate an answer without response options. Expected response length varied with the item’s cognitive demand, ranging from a single word or phrase to a brief explanatory statement, and was scored dichotomously (0 or 1 mark) using standardized model answers agreed upon by two subject experts (Supplementary File 2). MCQs followed the single-best-answer (Type A) format comprising one correct answer and four plausible distractors (Supplementary File 3).

All examination items were newly developed specifically for this study, independently reviewed by two Medical Parasitology experts, and validated for content. Disagreements were resolved by a third senior reviewer. Content validity was assessed using the Content Validity Index (CVI). The final blueprint ensured balanced representation of course content and cognitive levels. The passing mark for each exam was 50%.

To ensure the validity and defensibility of the passing threshold, a post-hoc Modified Angoff standard-setting method was employed. Two subject matter experts independently reviewed all 20 items and estimated the probability (ranging from 0.0 to 1.0) that a ‘borderline’ (minimally competent) first-year student would answer each item correctly. The consensus cut-off score was calculated as the sum of these probabilities, which empirically validated the institutional 50% passing threshold (10.0 out of 20 points) as a scientifically sound benchmark for this assessment (Table s2).

### Pilot testing

Draft items underwent pilot testing with a small group of students. A total of 20 SAQs and 20 MCQs representing the full range of LOs and cognitive levels were selected for piloting. Feedback was obtained by reviewing examinee responses regarding clarity, difficulty, and time allocation for each item. Two faculty reviewers from the Medical Parasitology Department independently observed the pilot session and recorded feedback.

### Examination procedures

The formative assessment was administered electronically at the end of the MLS-1 module. Students completed the SAQ examination first, followed immediately by the MCQ examination using the same content domains to prevent recognition of correct answers from influencing SAQ responses. Each examination consisted of 20 one-mark questions. Students were allocated 40 min for SAQs (2 min per question) and 20 min for MCQs (1 min per question). Questions were randomized individually for each student and presented one item per screen. The Qorrect platform operated within a secure browser-lockdown environment that prevented access to external resources while automatically enforcing examination timing. Student responses were continuously saved, and automatic reconnection protocols minimized data loss during temporary connectivity interruptions.

Standardized instructions were provided before the examination explaining the formative purpose of the assessment, examination procedures, and confidentiality. Students completed the examinations using their institutional usernames and passwords under direct supervision in computer laboratories. Randomized seating arrangements and electronic security measures minimized academic misconduct, while dedicated information technology staff remained available throughout the examination.

### Scoring and quality assurance

Each correct response received 1 mark, with no negative marking, resulting in total scores ranging from 0 to 20 for each examination. MCQs were scored automatically by the Qorrect platform using preloaded answer keys. Students received individualized electronic feedback including their scores and the correct responses for incorrectly answered questions. SAQs were independently scored by two blinded faculty examiners using standardized marking keys. Student identities were anonymized before marking through randomly generated identification codes. Discrepancies between examiners were resolved by consensus. Inter-rater agreement was evaluated using percentage agreement, with a predefined acceptable threshold of ≥ 80%. Additionally, 10% of anonymized scripts were randomly selected for review by a third senior faculty member to verify scoring consistency.

Following completion of both examinations, students participated in a structured feedback session addressing common misconceptions, examination performance, item clarity, cognitive demands, vignette quality, and time allocation. Students were encouraged to reflect on their reasoning processes, particularly for higher-order SAQs.

### Outcome measures

The primary outcome measures were P and DI.

The Difficulty Index represented the proportion of students answering each item correctly and was calculated according to Rezigalla et al. [5], as:

$$\mathrm{P}=\text{Number of students answering correctly}/\text{Total number of students}$$

Items were classified as difficult (0.00–0.30), moderately difficult (0.31–0.70), or easy (0.71–1.00).

The Discrimination Index measured each item’s ability to distinguish between high- and low-performing students using the upper and lower 27% of total examination scores and was calculated according to Rezigalla et al. [5], as:

$$\mathrm{DI}=\mathrm{(H-L)}/\mathrm{N}$$

where *H* represents the number of correct responses among the upper 27%, *L* the number among the lower 27%, and *N* the number of students in each group.

DI values were interpreted as excellent (≥ 0.35), acceptable (0.20–0.34), poor (0.00–0.19), or problematic (< 0).

Secondary outcomes included individual examination scores, item-level performance, comparisons between clinical vignette-based and factual questions, and psychometric characteristics across different student achievement levels.

### Statistical analysis

Student responses and psychometric indices were automatically generated by the Qorrect platform and exported to Microsoft Excel before statistical analysis.

Data were analyzed using IBM SPSS Statistics version 23.0 (IBM Corp., Armonk, NY, USA). Continuous variables were assessed for normality using the Shapiro–Wilk and Kolmogorov–Smirnov tests, along with inspection of histograms and Q–Q plots. Normally distributed data were summarized as mean ± standard deviation, whereas categorical variables were presented as frequencies and percentages. Paired-samples *t*-tests compared student scores, item difficulty indices, and discrimination indices between SAQs and MCQs. Independent-samples *t*-tests compared psychometric indices between high- and low-achieving students and between factual and clinical vignette-based items. McNemar’s test assessed agreement in pass/fail outcomes between examination formats. Pearson’s correlation coefficient examined relationships between item-level psychometric indices across assessment formats.

Effect sizes were reported using Cohen’s *d* and interpreted as small (0.2), medium (0.5), or large (0.8). All statistical tests were two-tailed, and statistical significance was established at *p* < 0.05.

## Results

### Participant characteristics and response rate

Of the 187 students enrolled in the Locomotor and Skin (MLS-1) module during the second semester of the 2024–2025 academic year, 178 (95.2%) completed both the short-answer question (SAQ) and multiple-choice question (MCQ) assessments and were retained for the final analysis. The remaining nine students (4.8%) were excluded because they did not complete both assessment formats.

### Test-level performance and reliability

Descriptive statistics for the two examination formats are summarized in Table 1. The MCQ examination produced a higher mean score than the SAQ examination (13.5 ± 4.5 vs. 11.1 ± 4.4 out of 20 points), corresponding to 67.5% and 55.5% of total marks, respectively. Both formats demonstrated excellent internal consistency (Cronbach’s α = 0.85 for MCQ, 95% CI [0.81, 0.89]; α = 0.83 for SAQ, 95% CI [0.79, 0.87]), well above the conventional acceptability threshold of 0.70. Mean item difficulty and discrimination indices were likewise higher for the MCQ (*P* = 0.67, DI = 0.44) than for the SAQ (*P* = 0.55, DI = 0.41), although average discrimination fell within the “good” range for both formats.

**Table 1:** Descriptive statistics of MCQ and SAQ examination performance (*N* = 178)

Measure	MCQ	SAQ
Mean	13.5	11.1
SD	4.5	4.4
Minimum score	0 (0%)	0 (0%)
Maximum score	20 (100%)	19 (95%)
Mean difficulty index (P)	0.67	0.55
Mean discrimination index (DI)	0.44	0.41

Grade distributions differed markedly between formats (Table 2): a substantially greater proportion of students fell in the Poor category (< 50%) on the SAQ (31.5%) than on the MCQ (20.8%), while a correspondingly larger proportion achieved Excellent grades on the MCQ (30.3%) than on the SAQ (11.2%).

**Table 2:** Grade distribution of students on MCQ and SAQ examinations (*N* = 178)

Grade	Score range	MCQ, *n*	MCQ, %	SAQ, *n*	SAQ, %
Excellent (A)	85–100%	54	30.3%	20	11.2%
Very Good (B)	75–84.9%	31	17.4%	27	15.2%
Good (C)	65–74.9%	24	13.5%	34	19.1%
Fair (D)	50–64.9%	32	18.0%	41	23.0%
Poor (F)	< 50%	37	20.8%	56	31.5%

Inter-rater reliability for SAQ scoring was high: two independent examiners achieved 94.2% exact agreement (95% CI [92.1, 96.3%]), exceeding the pre-specified ≥ 80% threshold [10]. The 5.8% of discrepant scripts were resolved by consensus discussion, and a third senior examiner’s review of a random 10% subsample confirmed 98.5% concordance with the consensus scores.

### Comparative analysis of MCQ and SAQ performance

Paired-samples comparisons (Table 3) confirmed that students scored significantly higher on the MCQ than on the SAQ examination (mean difference = 2.4 points; t(177) = 7.85, *p* < 0.001, 95% CI [1.8, 3.0], d = 0.59 — a medium effect). MCQ items were also significantly easier than SAQ items (mean difficulty difference = 0.12; t = 3.92, *p* < 0.001, 95% CI [0.04, 0.20], d = 0.71 — a large effect). By contrast, discrimination indices did not differ significantly between formats (t(19) = 0.86, *p* = 0.398, 95% CI [− 0.03, 0.09], d = 0.25), indicating that although the SAQ was the harder format, both formats discriminated between strong and weak students with comparable efficiency.

**Table 3:** Comparison of test-level performance and psychometric properties between MCQ and SAQ formats (*N* = 178 students; 20 items per format)

Measure	MCQ (M ± SD)	SAQ (M ± SD)	Mean diff.	t	*p*	95% CI	Cohen’s d
Total score	13.5 ± 4.5	11.1 ± 4.4	2.4	7.85	< 0.001	[1.8, 3.0]	0.59
Difficulty (P)	0.67 ± 0.15	0.55 ± 0.2	0.12	3.92	< 0.001	[0.04, 0.20]	0.71
Discrimination (DI)	0.44 ± 0.07	0.41 ± 0.11	0.03	0.86	0.398	[− 0.03, 0.09]	0.25
Reliability (α)	0.85	0.83	—	—	—	—	—

Paired-samples t-tests were used for continuous variables

Cohen’s d: 0.2 = small, 0.5 = medium, 0.8 = large

The total score comparison was conducted at the student level (df = 177, N = 178 students)

The difficulty (P) and discrimination (DI) comparisons were conducted at the item level (df = 19, N = 20 matched items)

*CI *Confidence interval

### Item-level difficulty and discrimination

The distribution of item-level indices (Table 4) showed that SAQ items were disproportionately concentrated in the difficult range (20% of SAQ items vs. 0% of MCQ items had *P* < 0.30), whereas MCQ items were more often easy (45% vs. 30%). Discrimination profiles were more comparable between formats, although a slightly greater proportion of SAQ items achieved very good discrimination (DI ≥ 0.50: 25% vs. 15% for MCQ).

**Table 4:** Distribution of item difficulty and discrimination indices (*N* = 20 items per format)

Category	MCQ, *n*	MCQ, %	SAQ, *n*	SAQ, %
Difficult (*P* ≤ 0.30)	0	0.0%	4	20.0%
Moderate (*P* = 0.31–0.70)	11	55.0%	10	50.0%
Easy (*P* > 0.70)	9	45.0%	6	30.0%
Fair (DI = 0.20–0.34)	3	15.0%	7	35.0%
Good (DI = 0.35–0.49)	14	70.0%	8	40.0%
Very good (DI ≥ 0.50)	3	15.0%	5	25.0%

Difficulty index (P) ranges from 0 (very difficult) to 1 (very easy)

Discrimination index (DI) ranges from − 1 to + 1; values ≥ 0.35 are considered excellent

Rows 1–3 classify items by difficulty; rows 4–6 classify the same items by discrimination

Item-level difficulty indices correlated strongly and significantly between the two formats (*r* = 0.71, *p* < 0.001; Table 5), indicating that items that were harder as SAQs tended also to be harder as MCQs. By contrast, the correlation between discrimination indices across formats was weak and non-significant (*r* = 0.01, *p* = 0.963), suggesting that an item’s discriminative power in one format did not reliably predict its performance in the other.

**Table 5:** Correlation between MCQ and SAQ item-level metrics (*N* = 20 items)

Metric	*r*	*P*	95% CI	Interpretation
Difficulty index (P)	0.71	0.001	[0.38, 0.87]	Strong, significant positive
Discrimination index (DI)	0.01	0.963	[-0.43, 0.45]	No significant

A full item-by-item comparison of all 20 items is provided in Table s1 (supplementary 4). Individual items illustrated both patterns identified above: Item 6 was markedly harder as an SAQ than as an MCQ (*P* = 0.20 vs. 0.53), yet Item 11 discriminated better in the harder SAQ format (DI = 0.69 vs. 0.52 for the MCQ). Conversely, Item 2 was much easier as an MCQ (*P* = 0.88 vs. 0.68), and Item 15 discriminated better as an MCQ despite that format’s greater ease (DI = 0.46 vs. 0.37 for the SAQ).

### Performance stratified by achievement level

**Fig. 1:**
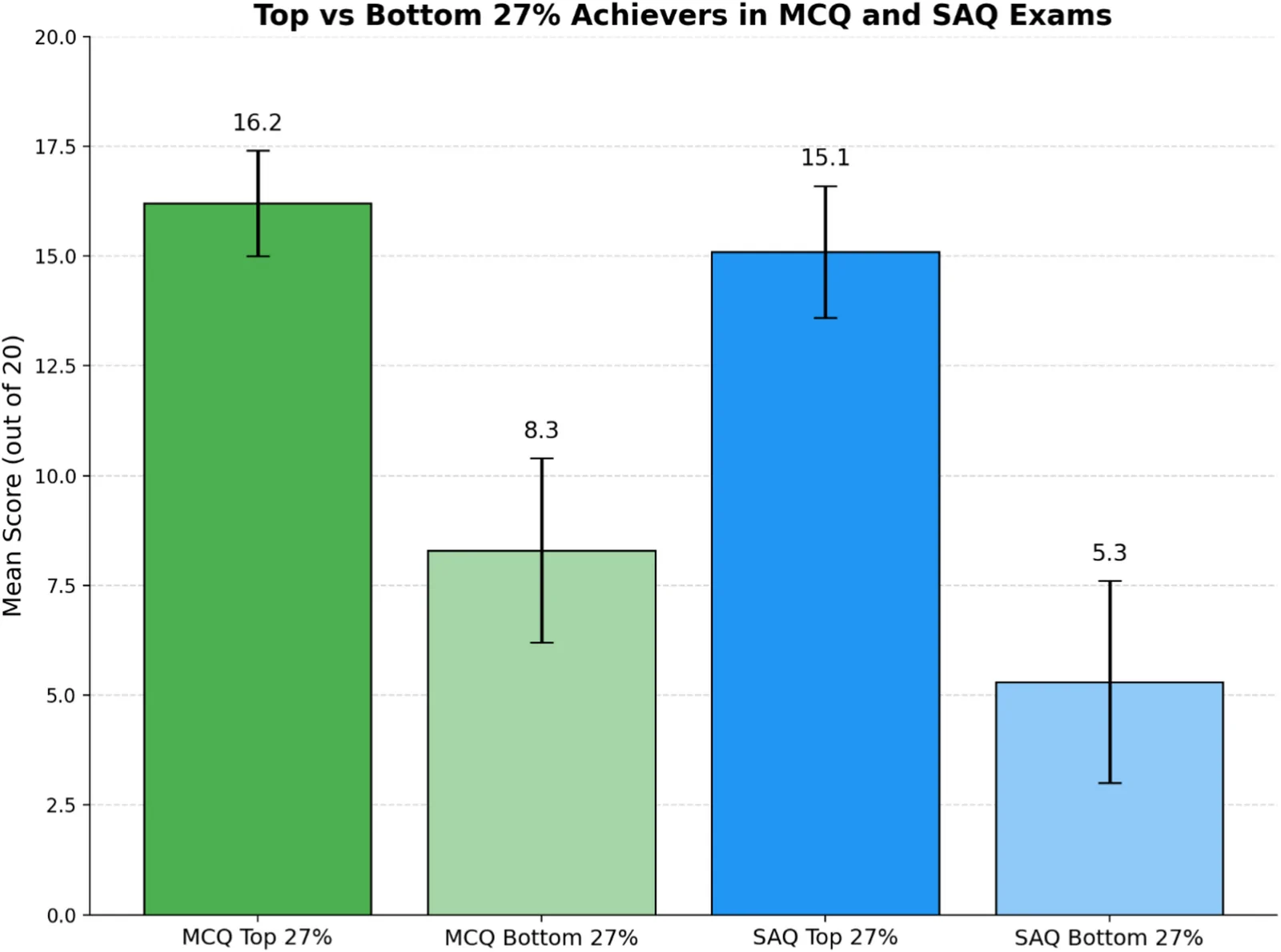
Pass/fail concordance between formats. Mean scores (± SD) of top 27% and bottom 27% achievers in MCQ and SAQ formats (*N* = 178). Error bars represent standard deviations. Both formats showed substantial separation between high and low achievers.

Students in the top and bottom quartiles (27%) of overall performance were compared within each format (Table 6; Fig. 1). Both formats significantly separated high from low achievers (MCQ: 7.9-point gap, t(94) = 22.6, *p* < 0.001, d = 4.6; SAQ: 9.8-point gap, t(94) = 24.7, *p* < 0.001, d = 5.0). The achievement gap was modestly larger for the SAQ than for the MCQ format.

**Table 6:** Comparison of top 27% vs. bottom 27% achievers on MCQ and SAQ formats (*N* = 178)

Group	MCQ (M ± SD)	SAQ (M ± SD)
Top 27% (*n* = 48)	16.2 ± 1.2	15.1 ± 1.5
Bottom 27% (*n* = 48)	8.3 ± 2.1	5.3 ± 2.3
Achievement gap	7.9 points	9.8 points
Within-format t-test (top vs. bottom)	t(94) = 22.6, *p* < 0.001, d = 4.6	t(94) = 24.7, *p* < 0.001, d = 5.0

Within-format comparisons used independent-samples t-tests (df = 94)

The achievement gap (MCQ: 7.9 points; SAQ: 9.8 points) is presented descriptively; a formal item-level test of discrimination equivalence between formats is reported in Table 3 (t(19) = 0.86, p = .398)”

Using a 50% passing threshold, 68.0% of students (*n* = 121) achieved concordant pass/fail outcomes across both formats (103 passed both, 18 failed both), while 32.0% (*n* = 57) showed discordant results (Table 7). Discordance was asymmetric: significantly more students passed the MCQ but failed the SAQ (21.3%, *n* = 38) than the reverse pattern (10.7%, *n* = 19; McNemar’s χ²(1) = 6.33, *p* = 0.012), reinforcing the finding that the SAQ was the more stringent of the two formats.

**Table 7:** Pass/fail agreement between MCQ and SAQ formats (*N* = 178)

Outcome category	*n*	%
Both formats passed	**103**	**57.9%**
Both formats failed	**18**	**10.1%**
MCQ passed, SAQ failed	**38**	**21.3%**
SAQ passed, MCQ failed	**19**	**10.7%**
Total	**178**	**100.0%**

Pass/fail outcomes were compared using McNemar’s test for paired categorical data

Passing threshold was ≥ 50% for both formats

### Performance by item type: factual knowledge vs. clinical vignettes

Items were classified as factual or clinical-vignette-based, and psychometric properties were compared within and across formats (Table 8). Within each format, factual and clinical items did not differ significantly in difficulty or discrimination (all *p* > 0.40). Across formats, factual items were significantly easier as MCQs than as SAQs (*P* = 0.7 vs. 0.57; t = − 2.29, *p* = 0.048, d = − 0.70, a medium-to-large effect), whereas the corresponding difference for clinical-vignette items (*P* = 0.65 vs. 0.52) was statistically significant (t = 3.15, *p* = 0.012, d = 1.00). Discrimination indices for both item types were statistically equivalent between formats (factual: *p* = 0.498; clinical: *p* = 0.540).

**Table 8:** Comparison of psychometric properties between factual and clinical-vignette items

Comparison	Factual (M ± SD)	Clinical (M ± SD)	t	*p*	d
Within SAQ — difficulty (P)	0.57 ± 0.21	0.52 ± 0.21	0.53	0.601	0.24
Within SAQ — discrimination (DI)	0.41 ± 0.12	0.42 ± 0.15	−0.26	0.794	−0.12
Within MCQ — difficulty (P)	0.7 ± 0.16	0.65 ± 0.15	0.72	0.480	0.32
Within MCQ — discrimination (DI)	0.43 ± 0.06	0.45 ± 0.08	−0.62	0.542	−0.29

Independent-samples t-tests compared item types within each format (rows above)

Format comparisons (SAQ vs. MCQ) within each item type are reported in the text: factual difficulty t = − 2.29, *p* = 0.048, d = − 0.70; factual discrimination t = − 0.70, *p* = 0.498, d = − 0.28; clinical difficulty t = 3.15, *p* = 0.012, d = 1.00 ; clinical discrimination t = − 0.63, *p* = 0.540, d = − 0.25

Cross-format correlation of discrimination indices, examined separately by item type (Table 9), was weak and non-significant for both factual (*r* = -0.11, *p* = 0.754) and clinical-vignette items (*r* = 0.08, *p* = 0.834), as well as for the pooled item set (*r* = 0.01, *p* = 0.963). This pattern suggests that the MCQ and SAQ formats may capture partially distinct competency dimensions regardless of item content.

**Table 9:** Cross-format correlation of discrimination indices by item type

Item type	*R*	*P*	*R*²	95% CI	Interpretation
Factual items (*n* = 10)	-0.11	0.754	0.01	[-0.66, 0.56]	No significant correlation
Clinical-vignette items (*n* = 10)	0.08	0.834	0.01	[-0.58, 0.67]	No significant correlation
Overall (pooled, *n* = 20)	0.01	0.963	0.00	[-0.43, 0.45]	No significant correlation

Finally, stratifying performance by achievement level (Table 10), high achievers performed comparably on factual and clinical items across both formats, whereas low achievers showed a more pronounced deficit on clinical-vignette items than on factual items — most notably in the SAQ format (mean *P* = 0.23 for clinical items vs. 0.30 for factual items). This finding suggests that weaker students found clinically contextualized, constructed-response items disproportionately challenging.

**Table 10:** Mean scores on factual and clinical-vignette items by achievement level

Format / Group	All items	Factual items (mean *P*)	Clinical items (mean *P*)
MCQ — all students (*N* = 178)	13.5 ± 4.5	7.0 ± 2.2	6.5 ± 2.3
MCQ — high achievers (*n* = 48)	16.2 ± 1.2	0.82	0.80
MCQ — low achievers (*n* = 48)	8.3 ± 2.1	0.43	0.40
SAQ — all students (*N* = 178)	11.1 ± 4.4	5.3 ± 2.6	5.7 ± 2.1
SAQ — high achievers (*n* = 48)	15.1 ± 1.5	0.79	0.72
SAQ — low achievers (*n* = 48)	5.3 ± 2.3	0.30	0.23
Overall (combined formats)	—	0.62	0.61

“All items” scores are reported as mean ± SD (raw points)

For achievement-level subgroups, mean P (difficulty indices) are reported to show the proportion of items answered correctly by each group. Ten factual and ten clinical-vignette items were included per format

## Discussion

This study systematically compared the psychometric properties of Short Answer Questions (SAQs) and Multiple-Choice Questions (MCQs) assessing identical learning outcomes in a Medical Parasitology course for first-year Egyptian medical students.

In medical education assessment, the terms short-answer question (SAQ) and very short-answer question (VSAQ) are used to describe related forms of constructed-response assessment, although terminology and definitions vary across the literature. Broadly, SAQs require learners to generate a response without answer options, with the expected response ranging from a brief phrase or sentence to a short, more elaborated response. VSAQs can be considered a more restricted form of constructed-response assessment, in which the expected response is typically limited to a word, phrase, number, or very brief statement [3, 7]. In the present study, the term SAQ is used operationally to refer to brief constructed-response items that required learners to generate answers independently, with expected responses ranging from single words or phrases to brief explanatory statements. Although some individual items had highly restricted response requirements that may overlap with the VSAQ format, the assessment was conceptualized and developed as a brief SAQ-based constructed-response assessment.

Our study demonstrates that MCQs and SAQs are effective tools for assessing item difficulty and their capacity to discriminate between low- and high-performing students. Three principal findings emerged: (1) SAQs demonstrated significantly higher difficulty than MCQs while maintaining comparable reliability and discrimination; (2) both formats showed excellent internal consistency (Cronbach’s α > 0.80) and strong ability to differentiate high from low achievers; and (3) despite mapping to the same intended learning outcomes, approximately 32.0% of students showed discordant pass/fail outcomes between formats, with significantly more students passing MCQs but failing SAQs. These findings may have important implications for assessment design in medical education and support ongoing efforts to improve the validity and comprehensiveness of student evaluation methods.

The current study found that at the test level, students achieved significantly higher scores on MCQs than on SAQs (67.5% vs. 55.5%, Cohen’s d = 0.59). This finding aligns with several previous studies in medical education. Palmer and Devitt [11] reported similar results among undergraduate medical students, in which MCQ performance exceeded SAQ performance across multiple content areas. They attributed this difference to the recognition-based nature of MCQs, which provides contextual cues that facilitate retrieval, whereas SAQs require unprompted recall without such cues.

Another study by van Wijk et al. [12] found that positive cueing was much more common in MCQs compared to open-ended very short-answer questions (VSAQs) (20% vs. 4%), where they assessed cueing empirically by comparing responses to content-matched MCQ and VSAQ items. Positive cueing was defined as correctly answering the MCQ while failing the equivalent VSAQ, indicating reliance on recognition cues provided by answer options rather than unprompted recall. Similarly, Mee et al. [13] found that MCQ formats tend to yield higher scores than constructed-response formats, reflecting differences in the cognitive processes involved. Newton et al. [14] further explained that MCQs may overestimate students’ competence because the format reduces the cognitive load required for answering.

The present finding of a 12% absolute difference in mean scores between formats supports this interpretation and suggests that MCQs alone may not fully capture the depth of student understanding. As Mee et al. [13] noted, generating the correct answer is generally more difficult than selecting it. Similarly, Pepple et al. [15] evaluated the performance of students in answering multiple-choice and essay questions and reported that the mean correct responses were 64% and 47%, respectively, indicating a significant difference between the two exam types.

In contrast, Bodkha [16] reported the opposite pattern among Indian medical students, where SAQs yielded higher scores (73.6% vs. 62.6% among high achievers). This discrepancy underscores that format difficulty is not intrinsic but context-dependent: item construction quality, time allocation per item, and grading stringency determine whether SAQs appear easier or harder than MCQs.

Despite differences in difficulty, both MCQs and SAQs exams demonstrated comparable discrimination indices (DI = 0.44 vs. 0.41; *p* = 0.398), indicating that both formats effectively differentiated between high and low performers.

Discrimination indices above 0.30 are generally considered indicative of good item performance, with values exceeding 0.40 reflecting very good discrimination [5]. Dhakal et al. [17] demonstrated that both MCQs and SAQs were capable of distinguishing between high- and low-performing students using ROC analysis, with SAQs showing slightly superior predictive performance. In their study, the discrimination index for structured short-answer questions (SSAQs) was 0.46, indicating very good item quality, whereas MCQs had a discrimination index of 0.29, which falls within the marginal range and suggests room for improvement. These findings from Dhakal et al. suggest that, in their context, SSAQs may be better at distinguishing between students of varying performance levels than MCQs.

Previous research has explored the comparability and objectivity of different assessment formats. Mehta et al. [18] reported a near-perfect correlation (*r* = 0.99) and strong agreement between MCQ and SAQ scores, concluding that carefully constructed short-answer questions can be as objective as multiple-choice questions. Although their study focused on score agreement and examiner objectivity rather than item discrimination, their findings support the broader notion that both formats can yield comparable overall assessment outcomes.

In the current study, both assessment formats demonstrated good internal consistency, with Cronbach’s α values of 0.85 for MCQs and 0.83 for SAQs; these values fall within the acceptable range of reliability coefficients (0.70–0.95) reported in the literature, with values above 0.80 generally indicating good internal consistency [19]. MCQs’ slightly higher reliability may be related to the standardized scoring process, which removes subjectivity. However, the SAQ grading process in the present study achieved 94.2% interrater agreement, indicating high consistency among raters.

Beyond reliability, content validity was ensured through the development of a detailed test blueprint aligned with the course’s intended learning outcomes. Both MCQ and SAQ formats were designed to assess identical learning outcomes across the same cognitive levels and content domains, ensuring proportional representation in accordance with instructional emphasis. This blueprint-guided approach improved comparability between assessment formats, as content-matched items targeted similar constructs. Consequently, any observed performance differences are less likely to be explained by variations in content and may instead reflect differences related to item format.

The present work also showed that, at the item level, a greater proportion of MCQ items were classified as easy compared with their SAQ equivalents )45% vs. 30%(.

The strong positive correlation in item difficulty across formats (*r* = 0.71, *p* < 0.001) indicates that the relative difficulty ranking of matched items was largely preserved between MCQs and SAQs. Items that were more difficult in one format tended to remain more difficult in the other. At the same time, the lower mean difficulty observed for SAQs suggests a consistent downward shift in performance associated with the response format. Together, these findings indicate that while item format influenced the overall level of difficulty, it did not appear to substantially alter the relative ordering of item difficulty across formats.

In contrast, the correlation between MCQ and SAQ discrimination indices was weak and not statistically significant (*r* = 0.01, *p* = 0.963), indicating that items did not consistently discriminate between high- and low-achieving students across formats. The presence of a strong correlation in difficulty alongside a weak correlation in discrimination suggests that MCQs and SAQs may not function as interchangeable measures, despite addressing the same intended content domain. Rather, they may reflect complementary aspects of student performance that are differentially influenced by response format.

In addition, the present study found significant discordance in pass/fail outcomes between MCQs and SAQs. While 68.0% of students achieved consistent outcomes, 21.3% passed MCQs but failed SAQs, compared with only 10.7% who demonstrated the opposite pattern (McNemar’s χ² = 6.33, *p* = 0.012). Although Mee et al. [13] did not examine pass/fail classifications, their finding that SAQs are consistently more difficult than MCQs in a high-stakes context may partly explain the discordance observed in the present study.

However, evidence from other settings suggests that such discordance is not universal. Saeed et al. [20] conducted a retrospective evaluation of second-year medical students’ physiology examinations at the University of Baghdad, analyzing 100 MCQ items and 100 essay-type questions drawn from the same final exam. Their analysis revealed no significant difference in overall pass rates between the formats, despite both formats assessing similar content domains. Similarly, the findings of the present study suggest that pass/fail decisions may be influenced by assessment format when based solely on MCQs.

The proportion of students passing MCQs but failing SAQs may indicate differential performance across response formats, potentially reflecting variations in recognition versus recall demands.

Within each assessment format, no significant differences were observed between factual and clinical vignette items in terms of difficulty or discrimination. However, when comparing across formats, factual MCQ items were slightly easier than factual SAQ items **(**0.7 vs. 0.57), with a marginally significant difference (*p* = 0.048), whereas a statistically significant difference was also found for clinical-vignette items (*P* = 0.65 vs. 0.52; *p* = 0.012), with MCQs being easier than their SAQ counterparts. Prior research has demonstrated that contextualization within MCQs can influence item difficulty.

Čapek et al. [21] assessed the impact of using clinical vignettes in MCQs on recall of factual pharmacology knowledge and found that context-rich vignette MCQs were answered correctly less often than factual questions. At the same time, other studies, such as Bosi et al. [22], noted that relevant clinical vignettes may improve discrimination and, in some cases, do not significantly affect item difficulty, highlighting that the impact of clinical context is highly dependent on item design.

Previous work by Norman [23] suggests that problem-solving tasks may reveal deficits in knowledge integration that are not captured by factual recall alone. While this was not directly demonstrated in the present study, the findings suggest the feasibility of incorporating vignette-based SAQs alongside other assessment formats. Furthermore, the viability of very short answer questions (VSAQs) as a practical and effective alternative to MCQs in medical student assessment has been increasingly supported in recent literature, offering the dual advantage of minimizing cueing effects while maintaining scoring feasibility in large cohorts [24].

### Educational implications

The findings of this study provide insight into how assessment format influences students’ knowledge retrieval performance within a formative module context. The observed differences in mean scores and pass/fail classification indicate that MCQs and SAQs may elicit different response patterns. Together, these results suggest the feasibility of employing mixed assessment formats to capture varied aspects of knowledge retrieval.

Students who passed MCQs but failed SAQs appeared to perform more strongly on recognition-based tasks than on constructed-response tasks requiring independent recall. Although clinical performance was not assessed, these findings suggest that incorporating SAQs alongside MCQs in formative assessments — and potentially in broader evaluation strategies — may offer additional insight into students’ ability to retrieve knowledge without cueing. Practical guidance for integrating such constructed-response formats into medical curricula has been outlined by Bala et al. [25], who emphasized the importance of clear marking rubrics, alignment with intended learning outcomes, and scaffolded introduction of VSAQ-style items to support student transition from recognition-based to recall-based assessment.

Both MCQ and SAQ formats demonstrated good discrimination indices, including vignette-based items, indicating the ability to differentiate between higher and lower performers within this cohort. These findings may support the inclusion of vignette-based items as part of a balanced assessment approach in similar educational contexts. However, the present study did not examine whether performance on these items predicts future academic outcomes or academic risk; therefore, such interpretations should be made cautiously.

Although the formative assessment was a scheduled, curriculum-integrated activity with a high participation rate, differences in students’ prior knowledge, preparation, motivation, and baseline ability may have influenced performance. In addition, the assessment was administered shortly before the high-stakes final examination, which may have increased students’ preparation and engagement. Therefore, test-taking behavior and performance observed in this formative context may not fully reflect performance under high-stakes summative conditions, and generalisation of the findings to other cohorts or educational settings should be made cautiously.

### Limitations

This study was conducted at a single institution among first-year medical students enrolled in a Medical Parasitology course, which may limit the generalizability of the findings to other settings, disciplines, or educational systems. Second, the assessment was not linked to external outcomes, such as subsequent academic or clinical performance, precluding evaluation of predictive validity. Third, the item-level correlation analyses were based on only 20 matched items. Although the observed correlation between MCQ and SAQ discrimination indices was essentially null (*r* = 0.01, *p* = 0.963), the relatively small number of items limits the statistical power to detect small-to-moderate associations. Fourth, all students completed the SAQ before the MCQ, without counterbalancing. Although this order was intentionally chosen to minimise the potential for MCQ response options to cue SAQ answers, it resulted in format and order being confounded. Therefore, differences between the two formats may partly reflect order-related effects, such as fatigue or changes in test-taking engagement, rather than format alone. Future studies should counterbalance the examination order or administer the two formats on separate occasions to better distinguish format effects from order effects. Finally, although the formative assessment was a scheduled, curriculum-integrated activity with a high participation rate, differences in students’ prior knowledge, preparation, motivation, and baseline ability may have influenced performance. In addition, the assessment was administered shortly before the high-stakes final examination, which may have increased students’ preparation and engagement. Therefore, test-taking behavior and performance observed in this formative context may not fully reflect performance under high-stakes summative conditions, and generalisation of the findings to other cohorts or educational settings should be made cautiously.

## Conclusions

Both multiple-choice questions (MCQs) and short-answer questions (SAQs) demonstrated satisfactory psychometric properties for formative assessment of first-year medical students. Although MCQs yielded significantly higher student scores and were associated with greater item ease, both formats showed comparable discrimination at the examination level and excellent internal consistency, supporting their reliability for assessing student performance.

The strong correlation between the difficulty indices of matched MCQ and SAQ items suggests that relative item difficulty was largely preserved across assessment formats. In contrast, the weak, non-significant correlation between discrimination indices indicates that individual items did not consistently differentiate high- and low-performing students in the same way. Furthermore, the observed discrepancies in pass/fail classification and the slightly greater difficulty of both factual and clinical SAQs compared with matched MCQs suggest that assessment format may influence both student performance and classification outcomes.

Taken together, these findings suggest that MCQs and SAQs should not be considered interchangeable assessment methods. Rather, each format appears to provide different and potentially complementary information about student performance. Whether combining the two formats provides additional educational or assessment benefit requires further investigation. Future studies involving larger, multicenter cohorts and additional medical disciplines are warranted to determine the generalizability of these findings and to examine whether the use of complementary assessment formats provides added value beyond either format alone.

## Supplementary Information

Supplementary Material 1.

Supplementary Material 2.

Supplementary Material 3.

Supplementary Material 4.

Supplementary Material 5.

## Data Availability

All data generated or analysed during this study are included in this published article and its supplementary information files.

## Abbreviations

CI: Confidence interval
CVI: Content Validity Index
DI: Discrimination index
P: Difficulty index
IT: Information technology
LO: Intended learning outcome
MCQ: Multiple-choice question
MLS-1: Locomotor and Skin module (first module)
ROC: Receiver operating characteristic
SAQ: Short answer question
SD: Standard deviation
SPSS: Statistical Package for the Social Sciences
SSAQ: Structured short answer question
VSAQ: Very short answer question
α: Cronbach’s alpha
