# Supplementary material for: Comparative analysis of short answer questions and multiple-choice questions in formative assessment of first-year medical students
Source: BMC Med Educ. 2026 Sep 23;26:1518. doi: 10.1186/s12909-026-10344-1 (PMC13602640; doi:10.1186/s12909-026-10344-1)
Supplement: Supplementary file 1 [file 12909_2026_10344_MOESM1_ESM.docx]

# **Supplementary 1-Blueprint**

**Course:** Medical Parasitology - Locomotor Module
**Academic Year:** 2024-2025
**Student Level:** First Year Medicine (5+2 Program)

**Course Intended Learning Outcomes (ILOs)**

By the end of this chapter, the student should be able to:

1. **List** the causative parasite
2. **Locate** the geographical distribution and recognize epidemiologic factors responsible for the prevalence of the disease
3. **Describe** the life cycle with special emphasis on infective stage, mode of infection, habitat and the hosts
4. **Correlate** the pathogenesis with the clinical picture & complications of the infection
5. **Outline** diagnostic methods and identify diagnostic specimens and stage
6. **Select** appropriate treatment
7. **Outline** a prevention and control plan

**Medical Parasitology Course - Locomotor Module Formative Assessment**

**Detailed Item-to-ILO Mapping**

**ILO 1: List the causative parasite (Remember - L1)**

| **Item** | **Format** | **Question Focus** | **Parasite** |
| --- | --- | --- | --- |
| 9 | SAQ/MCQ | Determine disease from clinical presentation | *Onchocerca volvulus* |
| 12 | SAQ/MCQ | Provide an example of anthroponotic transmission | *Leishmania tropica* |
| 16 | SAQ/MCQ | Recall key clinical feature or prevention | *Onchocerca volvulus* |
| 17 | SAQ/MCQ | Indicate species from Afghanistan case | *Leishmania tropica* |

**Bloom Level:** Remember (identifying/naming parasites)
**Items:** 3 items (15%)

**ILO 2: Locate geographical distribution & recognize epidemiologic factors (Remember/Understand - L1/L2)**

| **Item** | **Format** | **Question Focus** | **Geographic/Epidemiologic Element** |
| --- | --- | --- | --- |
| 13 | SAQ/MCQ | Identify species in chicle collectors, Central America | Geographic + occupational link |
| 20 | SAQ/MCQ | Predict transmission vector for Central American case | Geographic distribution of vector |

**Bloom Level:** Remember (geographic facts) + Understand (epidemiologic patterns)
**Items:** 2 items (10%)

**ILO 3: Describe life cycle (infective stage, mode of infection, habitat, hosts) (Understand/Apply - L2/L3)**

| **Item** | **Format** | **Question Focus** | **Life Cycle Component** |
| --- | --- | --- | --- |
| 3 | SAQ/MCQ | Predict diagnostic stage from culture | **Infective stage** (promastigote) |
| 4 | SAQ/MCQ | Identify mode of transmission | **Mode of infection** (sandfly bite) |
| 5 | SAQ/MCQ | Interpret vector breeding habitat | **Habitat** (swampy forested areas) |
| 20 | SAQ/MCQ | Predict transmission vector | **Vector** (*Lutzomyia*) |

**Bloom Level:** Understand (life cycle knowledge) + Apply (predicting stages/vectors)
**Items:** 4 items (20%)

**ILO 4: Correlate pathogenesis with clinical picture & complications (Understand/Apply - L2/L3)**

| **Item** | **Format** | **Question Focus** | **Pathogenesis-Clinical Correlation** |
| --- | --- | --- | --- |
| 1 | SAQ/MCQ | Explain myalgia in trichinosis | Larval migration to muscle |
| 2 | SAQ/MCQ | Explain Calabar swellings | Allergic/histamine response |
| 7 | SAQ/MCQ | Summarize cause of blindness | Microfilarial toxins + inflammation |
| 8 | SAQ/MCQ | Explain cause of death in trichinosis | Heart failure/pneumonia/encephalitis |
| 10 | SAQ/MCQ | Explain corticosteroid rationale | Reduce inflammatory response |
| 14 | SAQ/MCQ | Interpret negative Leishmanin test | Weak cell-mediated immunity |
| 15 | SAQ/MCQ | Explain lesion extension | Failure of CMI |
| 18 | SAQ/MCQ | Predict parasite from clinical syndrome | *Onchocerca* (vision loss + nodules) |

**Bloom Level:** Understand (explaining mechanisms) + Apply (clinical reasoning)
**Items:** 8 items (40%)

**ILO 5: Outline diagnostic methods & identify diagnostic specimens/stage (Understand/Apply - L2/L3)**

| **Item** | **Format** | **Question Focus** | **Diagnostic Element** |
| --- | --- | --- | --- |
| 3 | SAQ/MCQ | Predict stage found in culture | Promastigote stage |
| 11 | SAQ/MCQ | Contrast microfilaria features | Morphological identification |

**Bloom Level:** Understand (diagnostic principles) + Apply (identifying stages)
**Items:** 2 items (10%)

**ILO 6: Select appropriate treatment (Apply - L3)**

| **Item** | **Format** | **Question Focus** | **Treatment Aspect** |
| --- | --- | --- | --- |
| 6 | SAQ/MCQ | Predict corticosteroid role in trichinosis | Treatment rationale |
| 10 | SAQ/MCQ | Explain concurrent corticosteroid use with DEC | Combination therapy logic |

**Bloom Level:** Apply (selecting/justifying treatment choices)
**Items:** 2 items (10%)

**ILO 7: Outline prevention and control plan (Remember - L1)**

| **Item** | **Format** | **Question Focus** | **Prevention Strategy** |
| --- | --- | --- | --- |
| 19 | SAQ/MCQ | Recall prevention strategy for *Trichinella* | Meat inspection, proper cooking, freezing |

**Bloom Level:** Remember (recalling strategies)
**Items:** 1 item (5%)

**Assessment Blueprint**

| **Topic title** | **Bloom’s Levels** | **Items number** | **SAQs** | **MCQs** | **Item type CV\F** | **Bp%** |
| --- | --- | --- | --- | --- | --- | --- |
| **Trichinella spiralis**  **(muscle helminth)** | Remember, Understand,  Apply | 1, 6, 8, 19 | 4 | 4 | **CV:** 1  **F:**6, 8,19 | **20%** |
| **Loa loa** | Remember, Understand, | 2, 5, 11 | 3 | 3 | **CV:** 2  **F:** 5, 11 | **15%** |
| **Onchocerca volvulus** | Remember, Understand,  Apply | 7, 9, 10, 16, 18 | 5 | 5 | **CV:** 9, 10, 18  **F:** 7, 16 | **25%** |
| **Leishmania spp. (cutaneous protozoa)** | Remember, Understand, Apply | 3, 4, 12, 13, 14, 15, 17, 20 | 8 | 8 | **CV:** 3, 4, 15, 17, 20  **F:** 12, 13,14 | **40%** |
| **Total** | Remember (L1) 25%  Understand (L2) 40%  Apply (L3) 35% |  | **20** | **20** |  | **100%** |

Bp% = Percentage Weight of Each Topic in the Exam Blueprint

Clinical Vignette-Based (CV) = 10 items \ 20 (50%)

Factual Knowledge (F) = 10 items\ 20 (50%)
