# Supplementary material for: Comparative analysis of short answer questions and multiple-choice questions in formative assessment of first-year medical students
Source: BMC Med Educ. 2026 Sep 23;26:1518. doi: 10.1186/s12909-026-10344-1 (PMC13602640; doi:10.1186/s12909-026-10344-1)
Supplement: Supplementary file 2 [file 12909_2026_10344_MOESM2_ESM.docx]

# **Supplementary 2-SAQ Items**

| Item Num. | 1 |
| --- | --- |
| Item Stem | **A patient presents with myalgia with difficult mastication, periorbital edema, and splinter hemorrhages under the nails after consuming undercooked pork. What is the underlying cause of myalgia in this patient?** |
| Model Answer/Marking Guide | Larvae of *Trichinella spiralis* migrate to muscle. |

| Item Num. | 2 |
| --- | --- |
| Item Stem | **A traveler returns from a trip to West Africa complaining of a painless, temporary swelling on his forearm that disappears within a few days, only to reappear elsewhere. List one pathological mechanism of forearm swellings.** |
| Model Answer/Marking Guide | **Accept any of the following:**   - Localized hypersensitivity (allergic) reaction - Allergic reaction to migrating adult *Loa loa* - Histamine-mediated inflammatory response to migrating adult worms - Type I hypersensitivity to the migrating adult worm |

| Item Num. | 3 |
| --- | --- |
| Item Stem | **A patient from Iraq presents with indurated raised edge ulcers on upper limb with abundant discharge, as shown in the following image. Identify the developmental stage of this parasite that would be detected when culturing a scrape from the edge of the ulcer.**   |
| Model Answer/Marking Guide | Promastigote |

| Item Num. | 4 |
| --- | --- |
| Item Stem | **A patient presents with a slow-growing ulcer on the forearm after returning from a rural area in the Middle East. The lesion has a raised, indurated border with crusting.**  **Solve the given case scenario to identify one mode of transmission of this infection** |
| Model Answer/Marking Guide | **Accept any of the following:**  Bite of infected female sandflies.  Other, less common, modes of transmission include  congenital (transplacental) infection,  blood transfusion,  or accidental transmission to laboratory workers |

| Item Num. | 5 |
| --- | --- |
| Item Stem | ***Loa loa* transmission remains difficult to control due to the environmental breeding habits of its vector. State the breeding habitat of the vector responsible for this challenge.** |
| Model Answer/Marking Guide | Swampy areas of forests. |

| Item Num. | 6 |
| --- | --- |
| Item Stem | **Predict the main role of corticosteroids in the treatment of *Trichinella spiralis* infection.** |
| Model Answer/Marking Guide | To reduce the inflammatory/allergic reaction and prevent severe myocardial and CNS complications. |

| Item Num. | 7 |
| --- | --- |
| Item Stem | **State the main cause of blindness in *Onchocerca volvulus* infection.** |
| Model Answer/Marking Guide | **Acceptable answers:**   - Inflammation induced by microfilariae in ocular tissues. - Immune-mediated inflammation caused by microfilariae - Ocular inflammation due to microfilariae |

| Item Num. | 8 |
| --- | --- |
| Item Stem | **Identify the most common cause of death in complicated trichinosis.** |
| Model Answer/Marking Guide | **Acceptable answers:**  Heart failure (due to myocarditis)  Pneumonia  Septicemia  Encephalitis |

| Item Num. | 9 |
| --- | --- |
| Item Stem | **A patient has a travel history to tropical Africa with subcutaneous nodules, skin lesions, and ocular involvement.**  **Identify the most likely causative parasitic disease.** |
| Model Answer/Marking Guide | Onchocerciasis |

| Item Num. | 10 |
| --- | --- |
| Item Stem | **A 36-year-old man returns from a 6-month stay in rural West Africa with a sensation of something moving in his eye. The physician initiates treatment with diethylcarbamazine (DEC) and prescribes a corticosteroid concurrently.**  **Explain why corticosteroids are prescribed alongside DEC in in this patient** |
| Model Answer/Marking Guide | Reducing the inflammatory response caused by microfilarial death |

| Item Num. | 11 |
| --- | --- |
| Item Stem | **State one feature that distinguishes the microfilaria of *Loa loa* from that of *Onchocerca volvulus*.** |
| Model Answer/Marking Guide | **Acceptable answers:**  Sheathed  Nuclei extend to the tip of the tail  Found in peripheral blood during the day (diurnal periodicity) |

| Item Num. | 12 |
| --- | --- |
| Item Stem | **Provide an example of a parasite transmitted through anthroponotic transmission.** |
| Model Answer/Marking Guide | *leishmania tropica* |

| Item Num. | 13 |
| --- | --- |
| Item Stem | **Identify *Leishmania* species that is most commonly reported within chicle collectors in Central America** |
| Model Answer/Marking Guide | *leishmania Mexicana* |

| Item Num. | 14 |
| --- | --- |
| Item Stem | **A patient with diffuse cutaneous leishmaniasis has a negative Leishmanin skin test. Explain this finding.** |
| Model Answer/Marking Guide | due to weak or absent cell-mediated immune response in cases of diffuse cutaneous leishmaniasis |

| Item Num. | 15 |
| --- | --- |
| Item Stem | **A 28-year-old African male presents with multiple, non-ulcerative nodular lesions on his upper limbs and face, resembling lepromatous leprosy, that begin to extend to the rest of the body. Explain why the lesions start to extend.** |
| Model Answer/Marking Guide | due to failure of cell mediated immune response |

| Item Num. | 16 |
| --- | --- |
| Item Stem | **Describe cutaneous lesions in Sowda.** |
| Model Answer/Marking Guide | dark, thickened skin of one leg with papular rash |

| Item Num. | 17 |
| --- | --- |
| Item Stem | **A soldier work in Afghanistan develops a single, slowly enlarging dry ulcer on his cheek. Microscopy confirms Leishmania amastigotes. Indicate the most likely responsible species.** |
| Model Answer/Marking Guide | L.Tropica |

| Item Num. | 18 |
| --- | --- |
| Item Stem | **A 47-year-old man from a rural village in sub-Saharan Africa presents to the clinic with progressive vision loss and itching of both eyes. He reports a history of chronic skin itching and nodules over his trunk and arms. On examination, he has depigmented patches of skin, multiple firm subcutaneous nodules. Predict the most likely responsible parasite.** |
| Model Answer/Marking Guide | *Onchocerca volvulus* |

| Item Num. | 19 |
| --- | --- |
| Item Stem | **Recall one prevention strategy to minimize the risk of *Trichinella spiralis* infection.** |
| Model Answer/Marking Guide | proper inspection of pig's meat in slaughterhouses or  pork meat must be well-cooked with deep freezing of at -25°C for 10 days  or avoiding feeding raw garbage to pigs and combating rats |

| Item Num. | 20 |
| --- | --- |
| Item Stem | **A 35-year-old man from Central America is presented with the opposite ulcer. Predict how this infection is transmitted to humans.**   |
| Model Answer/Marking Guide | By the bite of an infected *Lutzomyia* sand fly |
