# Supplementary material for: Comparative analysis of short answer questions and multiple-choice questions in formative assessment of first-year medical students
Source: BMC Med Educ. 2026 Sep 23;26:1518. doi: 10.1186/s12909-026-10344-1 (PMC13602640; doi:10.1186/s12909-026-10344-1)
Supplement: Supplementary file 3 [file 12909_2026_10344_MOESM3_ESM.docx]

# **Appendix C-MCQ Items**

| Item Num. | 1 |  |
| --- | --- | --- |
| Item Stem | **A patient presents with myalgia with difficult mastication, periorbital edema, and splinter hemorrhages under the nails after consuming undercooked pork. Which of the following is the most likely underlying mechanism of the patient’s myalgia?** |  |
| ILO | student will be able to Correlate the pathogenesis with clinical symptoms and complication of infection |  |
| A | Embolic occlusion of local blood vessels |  |
| B | Larval encystment within striated muscle | * |
| C | Adult worm presence in tissue |  |
| D | Direct toxin release from adult |  |
| E | Enzymatic digestion of tissue by bacterial co-infection |  |

| Item Num. | 2 |  |
| --- | --- | --- |
| Item Stem | **A traveler returns from a trip to West Africa, complaining of a painless, temporary swelling on his forearm that disappears within a few days, only to reappear elsewhere. Which of the following is the most likely content of the forearm swellings?** |  |
| ILO | student will be able to *explain* the immunopathological mechanisms of loiasis |  |
| A | *Loa loa* larvae |  |
| B | *Onchocerca volvulous* larvae |  |
| C | *Onchocerca volvulous* adult |  |
| D | *Loa loa* adult |  |
| E | Histamine. | * |

| Item Num. | 3 |  |
| --- | --- | --- |
| Item Stem | **A patient from Iraq presents with indurated, raised-edge ulcers on the upper limb with abundant discharge, as shown in the following image. Which of the following figures represents the diagnostic stage found by culturing a scrape from the edge of the ulcer?**   |  |
| A |  . |  |
| B |  . |  |
| C |  . | * |
| D |  . |  |
| E |  . |  |

| Item Num. | 4 |  |
| --- | --- | --- |
| Item Stem | **A patient presents with a slow-growing ulcer on the forearm after returning from a rural area in the Middle East. The lesion has a raised, indurated border with crusting. Which of the following is the most likely mode of transmission of this infection?** |  |
| A | Consumption of undercooked meat |  |
| B | Bite of infected female sandfly | * |
| C | Direct contact with infected individuals |  |
| D | Bite of infected *Simulium* fly |  |
| E | Bite of infected Chrysops fly |  |

| Item Num. | 5 |  |
| --- | --- | --- |
| Item Stem | ***Loa loa* transmission remains difficult to control. Which of the following best explains why eradication efforts are challenging?** |  |
| A | The vector breeds in swampy, forested areas, making control difficult | * |
| B | The vector breeds with fast-flowing rivers or streams |  |
| C | The vector has a short lifespan, reducing exposure to insecticides |  |
| D | The vector is resistant to commonly used pesticides |  |
| E | The vector primarily inhabits dry desert regions |  |

| Item Num. | 6 |  |
| --- | --- | --- |
| Item Stem | **A patient diagnosed with *Trichinella spiralis* infection presents with severe myalgia, myocarditis, and CNS involvement. Corticosteroids are administered. What is the primary reason for this treatment?** |  |
| A | Directly kills larval stages of the parasite |  |
| B | Eliminates adult worms from the intestine |  |
| C | Reduces allergic and toxic immune reactions | * |
| D | Acts as a microfilaricidal agent |  |
| E | Prevents secondary bacterial infections |  |

| Item Num. | 7 |  |
| --- | --- | --- |
| Item Stem | **Which of the following is a major cause of blindness in *Onchocerca volvulus infection*?** |  |
| A | Female worms damage the optic nerve by physical pressure |  |
| B | Eggs of worms cause inflammation in the retinal layers |  |
| C | Microfilariae produce toxins and immunological responses. | * |
| D | larvae migrate to the brain, affecting optic nerve function. |  |
| E | A vector injects saliva containing toxins in the vertebral column |  |

| Item Num. | 8 |  |
| --- | --- | --- |
| Item Stem | **In complicated cases of trichinosis, death may occur. What is the most likely reason for this?** |  |
| A | Severe dehydration |  |
| B | Acute renal failure |  |
| C | Severe diarrhea |  |
| D | Heart failure | * |
| E | Severe anemia |  |

| Item Num. | 9 |  |
| --- | --- | --- |
| Item Stem | **A patient with a travel history to tropical Africa has subcutaneous nodules, skin lesions, and ocular involvement. Which of the following parasitic diseases is the most likely cause?** |  |
| A | Onchocerciasis | * |
| B | Myiasis |  |
| C | Trichinosis |  |
| D | Leishmaniasis |  |
| E | Loiasis |  |

| Item Num. | 10 |  |
| --- | --- | --- |
| Item Stem | **A 36-year-old man returns from a 6-month stay in rural West Africa with a sensation of something moving in his eye. He has started on diethylcarbamazine (DEC), and corticosteroids are prescribed concurrently.**  **Which of the following best explains the rationale for using corticosteroids with diethylcarbamazine in this patient?** |  |
| A | Reducing the inflammatory response caused by microfilarial death | * |
| B | Prevention of bone marrow suppression |  |
| C | Protection against reinfection during treatment |  |
| D | Reduction in gastrointestinal side effects |  |
| E | Enhancing the microfilaricidal efficacy of DEC |  |

| Item Num. | 11 |  |
| --- | --- | --- |
| Item Stem | **Which of the following is a key defining feature of the microfilaria of *Loa Loa* compared to *Onchocerca?*** |  |
| A | It has a sheath | * |
| B | It has no nuclei in the tail |  |
| C | It is found in blood at night |  |
| D | It does not appear in blood |  |
| E | It has smooth body curve |  |

| Item Num. | 12 |  |
| --- | --- | --- |
| Item Stem | **Which of the following *Leishmania* species is most closely associated with anthroponotic transmission?** |  |
| A | *Leishmania mexicana* |  |
| B | *Leishmania tropica* | * |
| C | *Leishmania braziliensis* |  |
| D | *Leishmania major* |  |
| E | *L. aethiopica* |  |

| Item Num. | 13 |  |
| --- | --- | --- |
| Item Stem | **Which of the following *Leishmania* species is most commonly reported among chicle collectors in Central America?** |  |
| A | *Leishmania mexicana* | * |
| B | *Leishmania tropica* |  |
| C | *Leishmania braziliensis* |  |
| D | *Leishmania major* |  |
| E | *L. aethiopica* |  |

| Item Num. | 14 |  |
| --- | --- | --- |
| Item Stem | **In which of the following conditions would you expect to see a negative Leishmanin skin test?** |  |
| A | *Leishmania braziliensis* leishmaniasis |  |
| B | Mucocutaneous leishmaniasis |  |
| C | Diffuse cutaneous leishmaniasis | * |
| D | Chronic cutaneous leishmaniasis |  |
| E | Acute Cutaneous Leishmaniasis |  |

| Item Num. | 15 |  |
| --- | --- | --- |
| Item Stem | **A 28-year-old African male presents with multiple, non-ulcerative nodular lesions on his upper limbs and face, resembling lepromatous leprosy, that begin to extend to the rest of the body. Which of the following parasites is the most likely cause?** |  |
| A | *Leishmania mexicana* |  |
| B | *Leishmania tropica* |  |
| C | *Leishmania braziliensis* |  |
| D | *Leishmania major* |  |
| E | *L. aethiopica* | * |

| Item Num. | 16 |  |
| --- | --- | --- |
| Item Stem | **The presence of a dark, thickened skin of one leg with papular rash in people of Yemen is suggestive of an infestation with which of the following parasites?** |  |
| A | *Leishmania major* |  |
| B | *Leishmania aethiopica* |  |
| C | *Loa loa* |  |
| D | *Onchocerca volvulus* | * |
| E | *Trichinella spiralis* |  |

| Item Num. | 17 |  |
| --- | --- | --- |
| Item Stem | **A soldier work in Afghanistan develops a single, slowly enlarging dry ulcer on his cheek. Microscopy confirms *Leishmania* amastigotes. Which species is the most likely responsible?** |  |
| A | *Leishmania mexicana* |  |
| B | *Leishmania major* |  |
| C | *Leishmania aethiopica* |  |
| D | *Leishmania tropica* | * |
| E | *Leishmania braziliensis* |  |

| Item Num. | 18 |  |
| --- | --- | --- |
| Item Stem | **A 47-year-old man from a rural village in sub-Saharan Africa presents to the clinic with progressive vision loss and itching of both eyes. He reports a history of chronic skin itching and nodules over his trunk and arms. On examination, he has depigmented patches of skin, multiple firm subcutaneous nodules. Which of the following parasites is most likely responsible?** |  |
| A | *Loa loa* |  |
| B | *Onchocerca volvulus* | * |
| C | *Trichinella spiralis* |  |
| D | *Leishmania braziliensis* |  |
| E | *Leishmania mexicana* |  |

| Item Num. | 19 |  |
| --- | --- | --- |
| Item Stem | **Which of the following is a potential prevention strategy to minimize the risk of *Trichinella spiralis* infection?** |  |
| A | Using broad-spectrum antibiotics |  |
| B | Freezing pork at –20°C for at least 10 days | * |
| C | Administering albendazole prophylactically to travelers |  |
| D | Wearing protective clothing to prevent insect bites |  |
| E | Washing fruits and vegetables thoroughly before eating |  |

| Item Num. | 20 |  |
| --- | --- | --- |
| Item Stem | **A 35-year-old man from Central America is presented with the opposite ulcer. Which of the following vectors is the cause of transmission?**   |  |
| A | *Simulium* |  |
| B | *Lutzomyia* | * |
| C | *Chryspos* |  |
| D | *Phlebotomus* |  |
| E | *Musca* |  |
