# Supplementary material for: Comparative analysis of short answer questions and multiple-choice questions in formative assessment of first-year medical students
Source: BMC Med Educ. 2026 Sep 23;26:1518. doi: 10.1186/s12909-026-10344-1 (PMC13602640; doi:10.1186/s12909-026-10344-1)
Supplement: Supplementary file 4 [file 12909_2026_10344_MOESM4_ESM.docx]

**supplementary 4**

**Detailed Item-by-Item Psychometric Analysis**

**Table s1.** Item-Level Difficulty and Discrimination Indices for All 20 Items

| **Item** | **Type** | **SAQ P-value** | **SAQ DI** | **MCQ P-value** | **MCQ DI** |
| --- | --- | --- | --- | --- | --- |
| l | Clinical | 0.55 | 0.37 | 0.56 | 0.53 |
| 2 | Factual | 0.68 | 0.29 | 0.88 | 0.38 |
| 3 | Clinical | 0.75 | 0.51 | 0.81 | 0.49 |
| 4 | Clinical | 0.43 | 0.44 | 0.55 | 0.32 |
| 5 | Factual | 0.54 | 0.30 | 0.59 | 0.49 |
| 6 | Clinical | 0.20 | 0.20 | 0.53 | 0.54 |
| 7 | Factual | 0.28 | 0.31 | 0.55 | 0.43 |
| 8 | Factual | 0.54 | 0.30 | 0.69 | 0.41 |
| 9 | Factual | 0.22 | 0.39 | 0.51 | 0.33 |
| 10 | Factual | 0.74 | 0.59 | 0.82 | 0.42 |
| 11 | Clinical | 0.84 | 0.69 | 0.87 | 0.52 |
| 12 | Clinical | 0.59 | 0.33 | 0.64 | 0.43 |
| 13 | Factual | 0.70 | 0.46 | 0.87 | 0.48 |
| 14 | Factual | 0.71 | 0.53 | 0.42 | 0.32 |
| 15 | Factual | 0.76 | 0.37 | 0.87 | 0.46 |
| 16 | Factual | 0.57 | 0.49 | 0.78 | 0.48 |
| 17 | Clinical | 0.37 | 0.50 | 0.73 | 0.46 |
| 18 | Clinical | 0.37 | 0.32 | 0.51 | 0.39 |
| 19 | Clinical | 0.30 | 0.39 | 0.48 | 0.40 |
| 20 | Clinical | 0.79 | 0.41 | 0.79 | 0.43 |

P-value = Difficulty index (0 = very difficult, 1 = very easy). DI = Discrimination index (–1 to +1, with ≥0.35 considered excellent). Type: Clinical = clinical vignette-based item; Factual = factual knowledge item.
