# Supplementary material for: Comparative analysis of short answer questions and multiple-choice questions in formative assessment of first-year medical students
Source: BMC Med Educ. 2026 Sep 23;26:1518. doi: 10.1186/s12909-026-10344-1 (PMC13602640; doi:10.1186/s12909-026-10344-1)
Supplement: Supplementary file 5 [file 12909_2026_10344_MOESM5_ESM.docx]

**Table s2: Post-hoc Modified Angoff Standard Setting Judgments**

| **Item No.** | **Item Type** | **Expert 1 Probability** | **Expert 2 Probability** | **Consensus Probability** |
| --- | --- | --- | --- | --- |
| 1 | Clinical | 0.50 | 0.52 | 0.51 |
| 2 | Factual | 0.60 | 0.64 | 0.62 |
| 3 | Clinical | 0.65 | 0.71 | 0.68 |
| 4 | Clinical | 0.38 | 0.42 | 0.40 |
| 5 | Factual | 0.48 | 0.52 | 0.50 |
| 6 | Clinical | 0.15 | 0.21 | 0.18 |
| 7 | Factual | 0.23 | 0.27 | 0.25 |
| 8 | Factual | 0.47 | 0.51 | 0.49 |
| 9 | Factual | 0.18 | 0.22 | 0.20 |
| 10 | Factual | 0.65 | 0.69 | 0.67 |
| 11 | Clinical | 0.74 | 0.78 | 0.76 |
| 12 | Clinical | 0.52 | 0.56 | 0.54 |
| 13 | Factual | 0.62 | 0.66 | 0.64 |
| 14 | Factual | 0.63 | 0.67 | 0.65 |
| 15 | Factual | 0.67 | 0.71 | 0.69 |
| 16 | Factual | 0.51 | 0.55 | 0.53 |
| 17 | Clinical | 0.32 | 0.36 | 0.34 |
| 18 | Clinical | 0.32 | 0.36 | 0.34 |
| 19 | Clinical | 0.26 | 0.30 | 0.28 |
| 20 | Clinical | 0.70 | 0.74 | 0.72 |
| **Total** |  |  |  | **9.99/20** |
| **Recommended cut score** |  |  |  | **49.95% ≈ 50.0% (10/20)** |
